# Sensing and Self-Sensing Actuation Methods for Ionic Polymer–Metal Composite (IPMC): A Review

**DOI:** 10.3390/s19183967

**Published:** 2019-09-14

**Authors:** WanHasbullah MohdIsa, Andres Hunt, S. Hassan HosseinNia

**Affiliations:** 1 Department of Precision and Microsytems Engineering, Faculty of Mechanical, Maritime and Materials Engineering, Delft University of Technology, 2628 CD Delft, The Netherlands; A.Hunt-1@tudelft.nl (A.H.); S.H.HosseinNiaKani@tudelft.nl (S.H.H.); 2 Faculty of Manufacturing and Mechatronic Engineering Technology, Universiti Malaysia Pahang, 26600 Pekan, Pahang, Malaysia

**Keywords:** IPMC, sensing, self-sensing, transducer, smart material, ionic polymer, electroactive polymer

## Abstract

Ionic polymer–metal composites (IPMC) are smart material transducers that bend in response to low-voltage stimuli and generate voltage in response to bending. IPMCs are mechanically compliant, simple in construction, and easy to cut into desired shape. This allows the designing of novel sensing and actuation systems, e.g., for soft and bio-inspired robotics. IPMC sensing can be implemented in multiple ways, resulting in significantly different sensing characteristics. This paper will review the methods and research efforts to use IPMCs as deformation sensors. We will address efforts to model the IPMC sensing phenomenon, and implementation and characteristics of different IPMC sensing methods. Proposed sensing methods are divided into active sensing, passive sensing, and self-sensing actuation (SSA), whereas the active sensing methods measure one of IPMC-generated voltage, charge, or current; passive methods measure variations in IPMC impedances, or use it in capacitive sensor element circuit, and SSA methods implement simultaneous sensing and actuation on the same IPMC sample. Frequency ranges for reliable sensing vary among the methods, and no single method has been demonstrated to be effective for sensing in the full spectrum of IPMC actuation capabilities, i.e., from DC to ∼100 Hz. However, this limitation can be overcome by combining several sensing methods.

## 1. Introduction

Smart material transducers provide novel solutions for actuation and sensing, allowing realization of designs that are beyond the capabilities of the conventional transducers. For example, electrostrictive [1] and piezoelectric [2] transducers are used for high-speed and high-precision positioning, and shape memory alloys are exploited in various temperature-adjusting structures [3]. Introduction of the mechanically compliant and high-strain-capable bending transducers, such as dielectric elastomer actuators (DEA) [4], piezopolymer transducers [5], and ionic polymer transducers (IPT) [6] has stimulated research in various fields, including soft robotics, biomedical engineering, small-scale robotics, and bio-inspired robotics [7]. The simple structure of these smart materials allows easy customization of them, in order to realize simple actuation and sensing solutions in otherwise complex designs [8].

**Figure 1:**
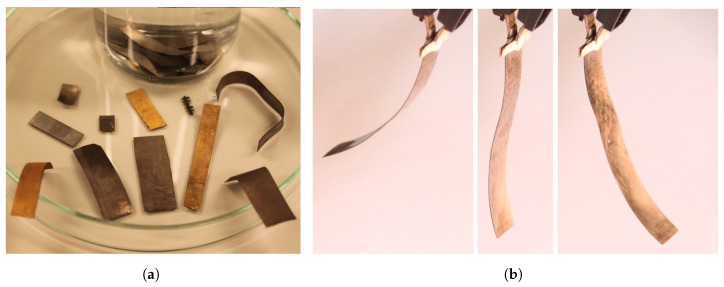
(**a**) IPMC samples of different types and geometries. (**b**) IPMC actuation: bending in response to application of 2.5 V input at opposite polarities (left and right), and IPMC at rest (middle).

**Figure 2:**
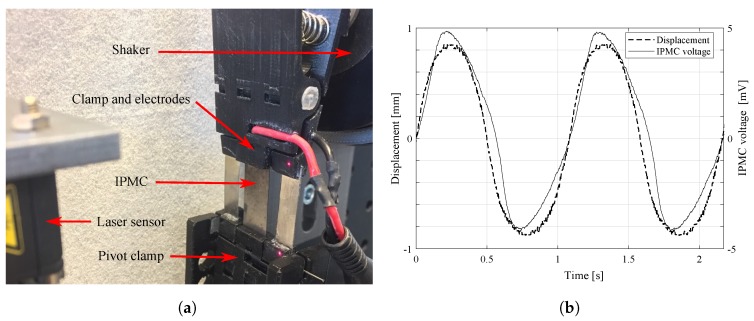
Implementation of IPMC sensing. (**a**) IPMC is mechanically excited using a shaker, while measuring the displacement and generated voltage. (**b**) Measured voltage readings in response to 1 Hz excitation.

Ionic polymer–metal composites (IPMC) are IPTs that are made by coating a thin sheet of ionic polymer with metal electrodes. These materials bend in response to low-voltage stimuli as shown in Figure 1 (typically <5 V amplitude), and generate voltage in response to bending as shown in Figure 2. They can be used for actuation, sensing, and self-sensing actuation (SSA). Since IPMCs operate at low voltages, they are simple to couple to electronics, and unnecessary to insulate. The latter also makes IPMCs simple to post-process, unlike DEAs and piezopolymers, whose insulation may not be compromised. However, their adaptation into practical use has been limited thus far by implementation challenges and complex dynamic behavior.

Besides electromechanical and mechanoelectrical transduction properties, IPMCs can also be used for self-sensing during actuation (SSA) [9,10,11,12,13]. Using IPMCs for sensing or self-sensing allows obtaining of deformation readings with significantly more compact, integratable, economic and lightweight design than with conventional transducers. IPMCs can replace bulky vision systems [14,15], laser displacement sensors [16,17], load cells [18,19] and inductive sensors [20] from actuation systems of smart materials. Proposed IPMC sensing applications include sensing of vibrations [21], seismic waves [22], position in multiple degrees of freedom [23], bio-acoustic waves [24], force [25], pressure [26,27], tactile interactions [28] and flow [29].

IPMC properties and potential applications have been investigated for more than two decades [30]. While sensing has received less attention than actuation, several methods have been investigated for using IPMCs as sensors and self-sensing actuators in a multitude of reports. Since interest in using IPMCs for sensing and SSA is increasingly growing, a summarizing study is needed to compile together the available information on implementation, characteristics, limitations of the available methods.

**Figure 3:**
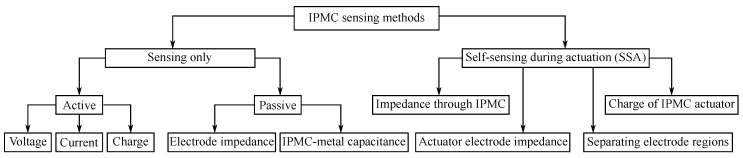
Classification of reported sensing and SSA methods for IPMCs.

This paper will review and analyze the reported methods for IPMC sensing and self-sensing actuation that use IPMCs for deformation sensing as illustrated in Figure 3. First, we will explain some fundamentals of IPMCs in Section 2, and summarize the efforts to model and elucidate the IPMC sensing phenomenon in Section 3. Next, classification of reported IPMC sensing methods into active, passive and self-sensing actuation (SSA) types will be explained in Section 4. Section 5 will review the active sensing methods, i.e., methods that rely on IPMC mechanoelectrical transduction. Section 6 will address passive sensing methods, measuring variations in impedances of the material itself, or in combination with external circuitry. Self-sensing actuation methods implementing sensing simultaneously with actuation will be reviewed in Section 7. Furthermore, we will discuss and compare IPMC sensing and SSA methods in terms of their behavior in frequency domain and other aspects in Section 8, and conclude the findings in Section 9.

## 2. Ionic Polymer–Metal Composite (IPMC)

Smart materials are material-level transducers that convert energy between physical domains [31], typically between mechanical and another domain, e.g., electric, electromagnetic [32], chemical [33], thermal [34], optical [35], etc. IPMCs are smart material transducers that exhibit both electromechanical (Figure 1) and mechanoelectrical (Figure 2) transduction properties, therefore making them two-way transducers [31]. They are obtained by coating ion-conductive electroactive polymer (a smart material) with electrically conductive electrodes [6].

**Figure 4:**
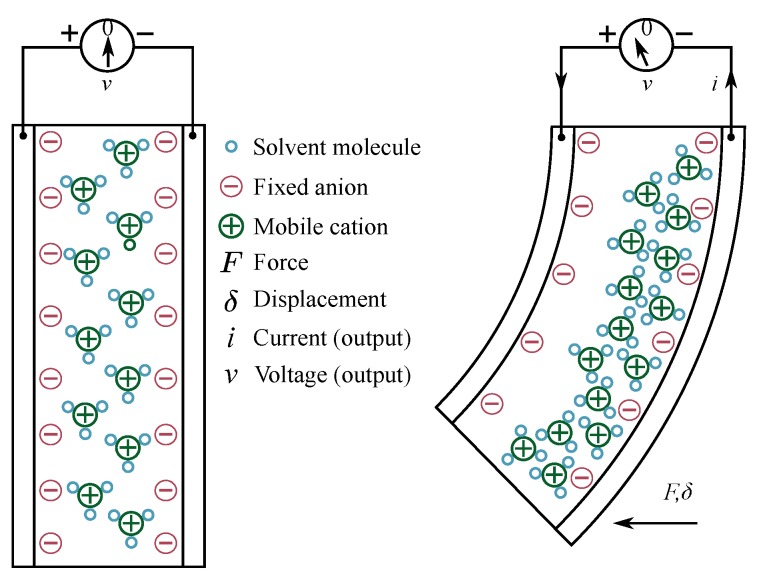
Sensing phenomenon inside IPMCs. Movement of mobile counter-ions causes accumulation of charge in the electrode boundary layers [47].

While IPMCs with different geometries have been reported, e.g., [36,37], they are most often manufactured as thin trilayer composite membranes, as illustrated in Figure 1 and Figure 4. They are typically 200μm thick, whereas the polymer backbone contributes 180μm (dry membrane) [38], the electrodes are approximately 10μm each [39], and extend their dendritic structures into the polymer [40]. Several different fabrication methods have been reported to produce the IPMC transducers. The most common technique is using a commercially available polymer membranes, e.g., [41], chemically plating them with Pt or Au electrodes, and ion-exchanging the loosely coupled ions for desired chemical species [42]. It is also possible to create custom backbones using solution recasting methods [43], 3D printing [37] hot-pressing [44] and spray-painting [45]. Electrodes can alternatively be deposited on the polymer by direct assembly process (DAP) [46].

Different ion-conductive polymer membranes can serve as the IPMC backbone, such as perfluorinate Nafion [48], Flemion [48], Selemion-CMV [49], non-perfluorinated Kraton [50], sulfonated polystyrene (SPS) [51] and polyvinylidene fluoride—polyvinyl pyrrolidone—polystyrene sulfonic acid (PVDF/PVP/PSSA) [52]. He et al. proposed doping Nafion with nitrogen-doped carbon nanocages [53] for improved transduction properties. Different metals can serve as IPMC electrodes, including platinum [54] (Figure 1), palladium [55], palladium—platinum [56], copper—nickel [57], gold [58] and silver [59]. Wang et al. used alcohol-assisted electroless plating, achieving enhanced transduction properties [44]. Alternatively, the polymer can also be coated by non-metal electrodes, such as carbon [60], but this case it is inaccurate to address these materials as “IPMC”. In order to further tailor the material actuation and sensing properties, the mobile (i.e., loosely coupled) counter-ions within the polymer backbone can be exchanged for other chemical species, e.g., alkali—metal cations Li${}^{+}$, Na${}^{+}$, K${}^{+}$, Rb${}^{+}$, and Cs${}^{+}$, as well as alkyl—ammonium cations, tetramethylammonium (TMA${}^{+}$) and tetrabutylammonium (TBA${}^{+}$) [61].

In terms of mechanical properties, IPMC Young’s modulus depends on material parameters such as ionomer type, counter-ions and solvent uptake [6,61,62,63,64,65], ranging from tens of MPa to several GPa, e.g., 20 MPa [6], 70–140 MPa [66], 70 MPa–1.4 GPa [63,64], 140–658 MPa [65], 160 MPa [67], 400 MPa–1.4 GPa [62], and 500–650 MPa [68]. Reported yield stresses and strains are approximately 5–50 MPa and 15%, respectively [69]. Detailed study on elastic behavior of different IPMC compositions can be found in [69].

Actuation phenomenon (Figure 1) in IPMCs is caused by unequal stresses within the opposite electrode boundary layers. It is caused by ion migration that occurs in IPMC thickness direction upon application of voltage, due to formation of electric field [6,70]. Similarly, IPMC sensing phenomenon (Figure 2) occurs due to migration of mobile counter-ions upon material deformation (see Figure 4). This causes charge imbalance in the material thickness direction that can be measured as voltage difference between the electrodes [6,70]. Reports disagree on the exact processes that dominate the actuation and sensing phenomena, and the hypothesized sensing mechanisms will be discussed with modelling in Section 3.

## 3. Models for IPMC Sensing

This section will introduce different modelling approaches to describe the sensing behavior of IPMC bending transducers. We will investigate the models for active sensing methods, i.e., models that relate the mechanical deformation of the material to its electrical response. Proposed black-box (empirical), grey-box (mix of empirical and physical) and white-box (physical) models will be addressed in the respective following subsections. Models for passive sensing and SSA are omitted since they mostly base on known implementations of impedance measurements.

### 3.1. Black-Box Models

Black-box models are direct representation of relationships between system’s input and output that can be constructed from experimental data using algebraic equations, with no prior knowledge about underlying physics [71,72]. They are simple to implement and identify, which makes them useful for practical engineering applications, but system-specific and unscalable, e.g., with dimensions of an IPMC sample. Black-box models have been intensively used to identify transfer function models of IPMC actuation dynamics, either for feedforward or feedback control purposes [16,73,74,75,76,77,78,79,80,81], while they have been used much less intensively to describe IPMC sensing dynamics.

In 2009, Hunt et al. [82] characterized IPMC sensing dynamics using a polynomial transfer function to achieve a stable and minimum-phase black-box sensing model. Its inverse dynamics was used to estimate the position of the tip of an integrated IPMC sensor-actuator design. While reliability of the estimated position reading reduced rapidly due to low-frequency noise, the IPMC position estimation produced much higher resolution than their camera-based external position measurement system.

In 2010 and 2011, Ganley et al. [83,84] modelled IPMC sensing with fourth order linear time-invariant transfer function models. These models described the dependence of IPMC sensing dynamics on temperature, in the frequency interval of 10 to 100 Hz. Their displacement trajectory estimation under sinusoidal and free vibration input proved comparable to the external laser sensor reading.

### 3.2. Grey-Box Models

Grey-box models base on partial understanding of physics that governs the system behavior, and compensate for the unknown parts with empirical models [71,72]. Therefore, they partially consist of white-box and partially of black-box models. Grey-box models provide a trade-off between essential needs to understand the system, with simplicity from the system identification point of view. In the following we will investigate the grey-box models that have been used to describe IPMC mechanoelectrical transduction.

**Figure 5:**
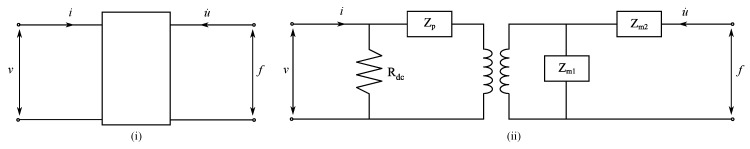
IPMC grey-box models: (i) two-port model concept [85,86], and (ii) transformer circuit model [87].

In 2002 Newbury and Leo [85] proposed using a linear two-port model [86] to describe both sensing and actuation dynamics of ionic polymer transducers, as illustrated in Figure 5. The model is written as

(1) $$\left(\begin{array}{c} v(\omega )\\ f(\omega ) \end{array}\right)=\left|\begin{array}{cc} Z_{11} & Z_{12}\\ Z_{21} & Z_{22} \end{array}\right|\;\cdot \;\left(\begin{array}{c} i(\omega )\\ \dot{u}\left(\omega \right) \end{array}\right),$$

where *v* and *i* stand for voltage and current on the IPMC electrodes, and *f* and $\dot{u}$ are respectively external loading force and the velocity at the free tip of the IPMC beam. $Z_{11}$ represents the electrical impedance of the immobile IPMC, $Z_{22}$ represents mechanical impedance of the electrically disconnected IPMC, while $Z_{12}$ and $Z_{21}$ are the electrical ↔ mechanical coupling terms. All the parameters in *Z* are empirical, and therefore this model is not scalable to IPMC sample dimensions. In 2003, Newbury and Leo proposed an improved equivalent circuit model, as shown in Figure 5 [87]. This model is based on an ideal linear transformer model [88], and the two-way electromechanical coupling of the IPMC is represented by turn ratio *N*, derived from governing equations of piezoelectrics. In the mechanical side of the model, $Z_{m1}$ and $Z_{m2}$ describe the relation between beam velocity and force. $Z_{m1}$ provides quasi-static Euler-Bernoulli beam equation relating the force on the beam to its velocity, and $Z_{m2}$ represents the beam dynamics. Electrical part models the electrical impedance of IPMC and consists of pure resistance $R_{dc}$, and dynamic impedance $Z_{p}$. This model captures both sensing and actuation dynamics, and is scalable. The model was later experimentally verified to well capture frequency domain behavior of the materials [89]. In [90] Akle and Leo adapted this model to describe the behavior of stacked IPMCs. Model structure was modified to account for electrical series and parallel connections, and mechanically the stacking effects were captured through boundary conditions at layer interfaces. Results showed that stacked IPMCs produce stronger sensing signal when electrically connected in parallel, and that stacking increases IPMC bandwidth at the expense of free displacement.

In 2006 Bonomo et al. [91] described IPMC current sensing by adapting a piezoelectric coupling model [88] and combining it with Euler-Bernoulli beam theory. Bode plots of their model identification results showed good correlation between the input and output signals within the frequency interval of 10–50 Hz, and the model was demonstrated to be potentially usable at frequencies of up to 150 Hz. It was also validated that the model is geometrically scalable. In 2008 Paola et al. improved accuracy of this model by considering the IPMC stiffness to be dynamic, and identifying the frequency-dependent Young’s modulus [21].

In 2010 Park and Yoon [92] compared several RC circuits to identify the most suitable equivalent circuit model to describe IPMC sensors. Mechanoelectrical coupling in these models was described by assuming that charge on the IPMC surface is linearly proportional to the bending angle. This study also included the equivalent circuit models of Shahinpoor et al. [93], Kanno et al. [94], as well as respective cascaded models. From simulations and experimental results it was concluded that the model of Kanno et al. [94] describes IPMC sensing most accurately. In 2012, Cha et al. [95] proposed an equivalent electrical circuit model for IPMC sensors as a parallel connection of a capacitor and a Warburg impedance that are further connected in series with a resistor. It was demonstrated that this model describes the IPMC sensing phenomenon more accurately than the previously reported RC equivalent circuit models.

### 3.3. White-Box Models

White-box models provide physical meaning to every part of the model. They describe the macroscopic behavior of a system by cascading the individual models of underlying sub-processes. White-box models for IPMC sensing describe the mechanoelectrical transduction through underlying mechanical, electrical, chemical and other processes that occur within the material. Compared to the modelling approaches described in Section 3.1 and Section 3.2, white-box models usually are more laborious to achieve, especially in explicit form, and are infrequently used in engineering application [72].

In 1995, Shahinpoor reported that IPMCs exhibit flexogelelectric effect, which is the principle phenomenon behind the active sensing methods in ionic polymer gels [96]. In 1998 Shahinpoor et al. hypothesized that the sensing effect is caused by shifting of the mobile charges within the material that occurs upon bending due to formation of stress gradient in the IPMC thickness direction [97]. Abstraction of this phenomenon is illustrated in Figure 4. In 2000, De Gennes et al. modelled the IPMC sensing phenomenon using a linear irreversible thermodynamic model in static conditions [98]. Their model described IPMC sensing through charge and solvent transportation within the polymer.

In 2000, Nemat-Nasser and Li [68] hypothesized that sensing phenomenon in IPMCs occurs due to the clustered topology of cations and anions within the material. Bending stresses were hypothesized to be caused by difference in displacements between the effective charge centers of cations and anions within each cluster, resulting in generation of electrical dipoles. These dipoles contribute to the formation of net electric field within the membrane. Constitutive equations were proposed to describe the processes that govern the actuation phenomenon of IPMCs, which later formed basis also for several physics-based IPMC sensing (and actuation) models. Proposed field equations relate the electric field E, electric displacement D, electric potential ϕ, charge density ρ, ion concentrations $C^{+}$ and $C^{-}$, and ion flux J within the polymer as follows:

(2) $$E=\frac{D}{\kappa_{e}}=\nabla \phi ,$$

(3) $$\nabla \;\cdot \;D=\rho =F(C^{+}-C^{-}),$$

(4) $$\frac{\partial C^{+}}{\partial t}+\nabla \;\cdot \;J=0,$$

where *F* is Faraday’s constant and $\kappa_{e}$ is the dielectric permittivity of the material, and J includes diffusion, migration and convection terms:

(5) $$J=-d\left(\begin{array}{c} \nabla C^{+}+\frac{C^{+}F}{RT}\nabla \phi +\frac{C^{+}\Delta V}{RT}\nabla p \end{array}\right)+C^{+}v,$$

whereas *d* is the ionic diffusivity, *R* is the gas constant, *T* is the absolute temperature, *p* is the fluid pressure and v is the is free solvent velocity. These sensing and actuation models agreed with the experimental observations that the voltage generated upon bending an IPMC is two orders of magnitude lower than the voltage required to produce actuation of the same amplitude.

Building on the above work [68,99], Farinholt and Leo (2004) [100] and Farinholt (2005) [101] reported a sensing model for IPMCs in electrically short-circuited configuration. The study proposed a linear partial differential equation for charge dynamics within IPMC, known as the Poisson–Nernst–Planck equation:

(6) $$\frac{\partial \rho }{\partial t}-d\frac{\partial^{2}\rho }{\partial x^{2}}+\frac{F^{2}dC^{-}}{\kappa_{e}RT}\left(1-C^{-}\Delta V\right)\rho =0$$

where the terms are consistent with Equations (2) to (5), and ΔV is the volumetric change. They hypothesized that charge density at the surface of the material is proportional to mechanical stress induced by bending.

In 2004 Konyo et al. [102] used IPMCs for velocity sensing, and for hypothesized that voltage generation upon bending is caused by two factors: (i) cation and solvent migration due to compression and expansion on the opposite faces of the IPMC; and (ii) inertial force acting on the counter-ions. They modelled IPMC as a parallel plate capacitor, and considered charge generation to be proportional to the beam velocity, which was further estimated using the governing equation for beam’s free vibrations.

In 2007 [103] and 2009 [104] Chen et al. further elaborated the work of [68,100,101], and reported a white-box transfer function model for IPMC sensors that is usable for real-time sensing and feedback control applications. This physics-based model was achieved by analytically solving Equation (6) in Laplace domain. The model also considered the distributed surface resistance effects. The model was reduced to a fourth order transfer function, and good agreement with experimental results was demonstrated.

In 2008 Porfiri [105] adapted multiphase mixture theory to establish a physics-based model for IPMC sensing and actuation. He derived an equivalent piezoelectric bimorph plate model for IPMCs to describe their behavior at static deformations, accounting for IPMC thickness, elastic properties, hydration level, solute concentration and permittivity. It was shown that the reduced order model behavior is in good agreement with experimental results.

Bahramzadeh and Shahinpoor (2011) [106] derived a model to relate the voltage generated by an IPMC sample to its curvature. The model was achieved by solving the Poisson–Nernst–Planck equation for ion diffusion (Equation (5)) in material thickness direction, and contains frequency-dependent coefficients to better capture the sensing dynamics.

Aureli and Porfiri (2013) [70] used the matched asymptotic expansions method to describe the boundary layers formation within the IPMC membrane and solved the Poisson–Nernst–Planck equation (Equation (5)) analytically, obtaining improved charge dynamics description. The electrical behavior of the IPMC was described using an equivalent circuit model that comprises of a series connection of a voltage source, a resistor, and two non-linear capacitors. Model was validated by comparing the simulation results against experimental results reported in previous reports. Lei et al. cascaded the model of ion transportation with base-excited beam model that has a mass in its tip, in order to describe IPMC sensing dynamics [107]. The model was simplified using Pade approximation method and Taylor series expansion to implement it in real-time structural vibration monitoring.

In 2014, Cha and Porfiri reported a chemoelectromechanical model for IPMC actuation and sensing at large quasi-static deformations [108,109]. The model based on polyelectrolyte gels modelling approach [110]. Components for the Poisson–Nernst–Planck equation were obtained using Helmholtz free energy density approach, accounting for the mechanical stretching, ion mixing and electrical polarization. Presuming perfectly conductive electrodes, semi-analytical model was derived to describe IPMC voltage sensing at small static deformations. Modelling results were validated against previously reported experiments, and it was estimated that voltages for sensing and actuation differ by 5 orders of magnitude.

Zhu et al. (2016) presented a dynamic multi-physics sensing model for IPMCs, and solved it for step displacement input [111]. This model provided a more detailed elaboration on the effects of transportation of both counter-ions and solvent on sensing. The model included convection under pressure gradient, ion migration in electric field, and inter-coupling between counter-ions and solvent. It was experimentally validated to well describe the sensing phenomenon, capturing both the fast rise and slow decay of the induced voltage in response to step deformation input.

In 2016 Lei et al. [23] adapted the works in [68,103] to model their tubular omnidirectional IPMC sensor. For implementation purposes, the model was further reduced to finite order using Pade approximation method. The original and reduced order models were respectively demonstrated to be able to estimate the sensing dynamics in frequency and time domains.

Based on [108,109], Volpini et al. (2017) derived a one-dimensional model for IPMC compression sensing [112]. The model was solved analytically and validated against finite element modelling results. It was reported that inhomogeneities within the polymer membrane are essential for compression sensing. Modelling results for IPMC current generation in response to compression were validated against experimental results in [113]. In 2019 Leronni and Bardella combined the modelling framework in [108,109] with sandwich beam theory [114] and zigzag warping model [115] in order to account for shear deformations in static sensing of IPMC bending [116]. Reportedly, the shear effects are negligible in thin IPMCs, but become more significant in short IPMC with stiff electrodes.

While IPMC sensing properties are well known to depend on material-specific characteristics, such as hydration level [117], cation species [101,118], solvent type [101] and dimensions [119], their response has been also reported to depend on ambient conditions, such as temperature [83,84], relative humidity [120,121,122,123], and potentially other factors.

## 4. Organizing IPMC Sensing Methods

Sensing methods for estimating bending state of IPMCs can be divided into active, passive, and SSA (self-sensing actuation) types. In case of such classification the active IPMC sensing methods rely on IPMC mechanoelectrical transduction, i.e., generation of electrical energy in response to bending. Passive IPMC sensing methods obtain information about material mechanical deformations by passing externally generated signals through (parts of) the IPMC sample, and measuring alterations in this signal due to variations in material electrical properties upon bending. SSA methods for IPMC sensing aim for realizing sensing functionality within the actuating material sample. Reported active and passive methods are summarized in Table 1, and SSA methods in Table 2. These methods are respectively addressed in Section 5 (active), Section 6 (passive) and Section 7 (SSA). Besides the measuring circuitry, sensing methods differ by complexity and robustness of implementation, achievable bandwidths, signal strengths, signal-to-noise ratios (SNR), etc. These characteristics are important to know when considering IPMCs for sensing applications.

## 5. Active Sensing Methods

Reported active methods for sensing IPMC deformations can be separated into three categories: (1) measuring voltage between IPMC electrodes (i.e., at high input impedance); (2) measuring current between IPMC electrodes in virtual short-circuit configuration (i.e., at near-zero loading impedance); and (3) measuring charge that is displaced between IPMC electrodes in virtual short-circuit configuration. Measurement circuitry provides electrical loading on the IPMC sample, converts the signal to voltage (if required), provides filtering if needed, and amplifies it to measurable levels for data acquisition. Reported methods virtually load IPMCs with either very low or very high input impedance. While impedance matching could be used to maximize energy transmission, it only been studied in IPMC energy harvesting [148] and telecommunication [149] studies, and has not been employed for sensing. In the following we will address the reported efforts and characteristics of these active sensing methods in more detail.

### 5.1. Voltage

**Figure 6:**
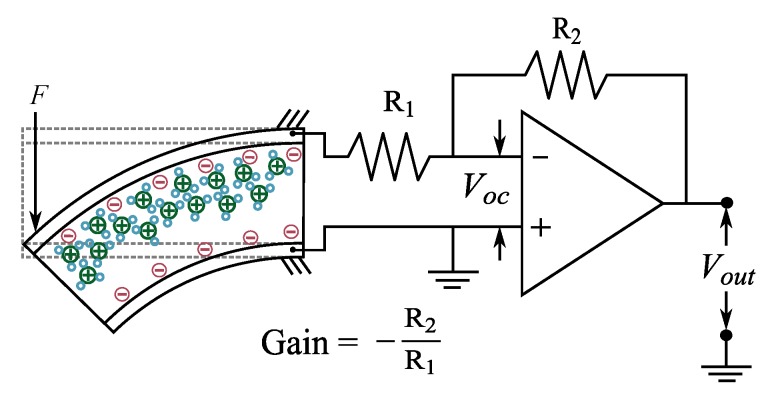
Implementation principle of IPMC voltage sensing.

IPMCs produce potential difference between their electrodes in response to bending deformations [150], and this phenomenon can be directly used for sensing purposes. Physical processes that cause this phenomenon were discussed with white-box models in Section 3.3. Implementation of IPMC voltage sensing is straight-forward: a voltage measurement circuit with high input impedance is coupled to IPMC electrodes, allowing the measurement of voltage between its electrodes while avoiding influencing the sample with electrical loading. Since the measurable voltages are very low, the first stage of sensing circuitry is usually an amplifier. Figure 6 shows a typical implementation that employs an inverting amplifier configuration.

#### 5.1.1. Reported Studies

First reports on voltage generation in platinized Nafion membranes in response to deformations date back to 1992. Sadeghipour et al. used a platinized Nafion membrane to measure pressure in a hydrogen pressure cell, and proposed an accelerometer design that consists of a Nafion membrane that is deformed between two contacting electrodes [150]. The designs were experimentally shown to generate voltage in response to the mechanical stimuli. Since then, a multitude of studies have investigated sensing the IPMC deformations by measuring voltage.

In 1995, Shahinpoor studied the “flexogelectric” effect, i.e., generation of electric field (and thus voltage) in non-homogeneously deformed ionic gels, which are in close contact with copper or platinum foil electrodes [96]. He theoretically and experimentally showed that the magnitude of the generated voltage is in the range of tens of millivolts. In 1997, Mojarrad and Shahinpoor investigated quasi-static displacement sensing with IPMCs, using an amplifier with the gain of 17.3 [124]. They observed capacitive behavior, and in sensing hysteresis study demonstrated linear behavior until the last quarter of the bending cycle. In 1998 Shahinpoor et al. extended this work by investigating IPMC response to impact loading [97]. They reported that IPMC responds with a well-repeatable damped voltage output, with bandwidth of up to 100 Hz. This was also reported by Shahinpoor et al. in their 2001 study on IPMC sensing dynamics [93]. In quasi-static experiments a slow current leakage through the material and presence of a small offset voltage were observed. It was hypothesized that properly removing the offset voltage would allow use of IPMCs for sensing DC deformations. IPMC electrical behavior was reported to be dominantly capacitive at low frequencies, and resistive above 100 Hz. Similar conclusions on IPMC electrical behavior were reported in the same year by Henderson et al. [117]. They studied usability of IPMCs as low-frequency accelerometers, and measured their voltage in response to vibrations in the frequency interval of 0.015 Hz to 50 Hz. IPMC response was observed to depend on the sample geometry and environmental humidity. They concluded that a dehydrated IPMC sample is usable as an accelerometer in sub 1 Hz applications, and it generates high enough voltage to be measurable without any amplifying circuitry.

In 2002, Newbury characterized the sensing dynamics of IPMC transducers, and reported that their voltage-displacement sensitivity is significantly inferior to that of PZT and PVDF transducers [125]. In 2003 Bonomo et al. studied IPMC voltage sensing properties using an amplifier with gain of 270, and reported that the voltage reading is dependent on the material hydration level and excitation frequency. They also observed the presence of time delay, and that response is non-linear, especially at low frequencies [130]. Konyo et al. (2004) reported that voltage on IPMC electrodes is proportional to its tip velocity, and proposed to pattern IPMC electrodes to achieve a 3DOF tactile sensor [102]. In 2006, Biddiss and Chau investigated feasibility of IPMC voltage sensing for hand prosthesis applications [126]. In 2009, Van Den Hurk et al. [132] and Chew et al. [131] proposed to use IPMCs for sensing joint rotations in robotic fingers. They observed a sequence of rapid increase and slow decay in IPMC voltage in response to bending, and estimated the bending speed and angle from the time and amplitude of the voltage peak. Later, McDaid et al. studied the same concept for their compliant surgical tool design [151]. Park and Yoon (2010) studied RC models for estimating bending angle from IPMC voltage [92] without implementing high-pass filters in signal conditioning [127]. In 2011, Bahramzadeh and Shahinpoor [106] studied using IPMCs for dynamic curvature sensing, and used a band pass filter (0.01 Hz to 10 Hz) with an amplifier (gain 10) to condition the IPMC voltage. They observed some frequency-dependence in response to sinusoidal deformations, and a voltage-recovery effects in case of step stimuli. In 2015, Gonzalez and Lumia measured the voltage generated by an IPMC beam for sensory feedback in their two-finger IPMC micro-gripper design [152].

In 2016, Zhu et al. studied IPMC sensing at different ambient humidity levels [123], and reported that humidity level strongly influences the magnitude and duration of the measured voltage response upon step deformations, as previously observed in [131,132,153]. In [118], Zhu et al. investigated the effect of cation species and water content on IPMC voltage generation. They reported that under step bending input, the amplitude of the voltage peak shows insignificant correlation to cations species, behaving similar to [131,132]; however, decay of the voltage response decreases with ambient humidity and becomes almost unobservable at 20% RH. Also, effect of humidity levels on sensing dynamics was reported for 0.1 Hz to 10 Hz frequency interval. Similar results were also reported in their modelling study [111].

Dominik et al. (2016) proposed connecting multiple IPMC samples in series or in parallel to improve the quality of the IPMC voltage sensing signal [154]. Experimental results in 1 Hz to 17 Hz frequency range showed that both the parallel and series connection result in higher sensitivity and lower noise than the individual IPMC samples. In 2017, Song et al. demonstrated on a 1 mm thick IPMC sample that encapsulating it in Parylene increases its usability interval as sensor in open air, since it slows down water evaporation [155]. Fu et al. (2018) showed that an IPMC sample manufactured by plasma etching the polymer and magnetron sputtering the electrodes generates close to 200 mV output in response to 1.6% bending strain, being 63 times higher than an electroless-plated IPMC [156]. In 2018, Khmelnitskiy et al. studied characteristics of IPMC voltage sensing both in air and in water environment using Pt-coated IPMCs with Nafion 117 membrane in Cu${}^{2+}$ cation form [129]. Sensitivity drop was reported at frequencies around 20 Hz and above, and authors argued that it is caused by slow motion of liquid within the polymer membrane.

In 2019, Wang et al. investigated the influence of geometry on IPMC sensing, concluding that thicker and shorter IPMCs exhibit higher voltage amplitudes, while the effect of width is insignificant. This contrasts with [155], where it was claimed that wider sensors display improved stability, while sensor thickness and length do not affect sensing magnitude. This difference may be caused by differences in sample surface conductivities. Zhu et al. proposed IPMC pressure sensors with the shape of a square frustum, and studied the effect of dimensions on their sensing performance [157]. Top and bottom surfaces of the frustum were plated with electrodes, and sensitivity increase was expected due to stress difference between the top and bottom electrode boundary layers. It was experimentally confirmed that sensitivity increases (first rapidly, and further slowly) with increasing difference between top and bottom electrode areas (2 mV/N for 1/1 and 10 mV/N for 1/7 area ratio), but does not change with the change in IPMC thickness.

MohdIsa et al. compared active IPMC sensing methods (i.e., voltage, current and charge) in terms of their frequency responses, coherences, and signal-to-noise-ratios within the 0.08–60 Hz frequency interval [133]. Bending deformations were constrained to first modal shape, and consistent displacements of 1.8 mm peak-to-peak were used in all experiments. Results showed that measuring IPMC voltage is feasible for sensing applications above ∼1 Hz, and that coherence and signal-to-noise ratio deteriorate at lower frequencies.

#### 5.1.2. Sensing Characteristics

When the free tip of an IPMC beam is deflected (i.e., step deformation), the voltage reading between its electrodes will first show rapid rise up to a peak value, and is then followed by a recovery phase towards initial static reading [106,126,131,132]. The peak voltage was shown to be approximately proportional to the bending angle for bending rates of 45–90° s${}^{-1}$, and voltage rise rate proportional to the bending rate [126]. Typical amplitudes of the voltage response remain in milli-volt range [97], e.g., 2.06 mV/mm (quasi-static) [97], 0.04 mV/mm (5 Hz) [125], 0.125 mV/mm (2 Hz) [106], 0.15 mV/mm (5 Hz) [118], and 0.072 mV/mm (10 Hz) [129]. Nemat-Nasser and Li showed by modelling and experimental data that the amplitude of IPMC sensing voltage is two orders of magnitude smaller than the voltage that is required to produce the same deformation in actuation [68]. The relatively small voltage amplitude has been hypothesized to be due to high capacitance of the material (2–5 mF/cm${}^{2}$) [158]. Several authors also reported offsets in the voltage reading [93,106,127], which are hypothesized to originate from the polyelectrolyte nature of IPMC [93]. Thicker, shorter and well-hydrated IPMCs exhibit higher amplitude in voltage reading [117,125,129,139], and sensing stability in atmosphere can be improved by coating the IPMC with Parylene [155].

**Figure 7:**
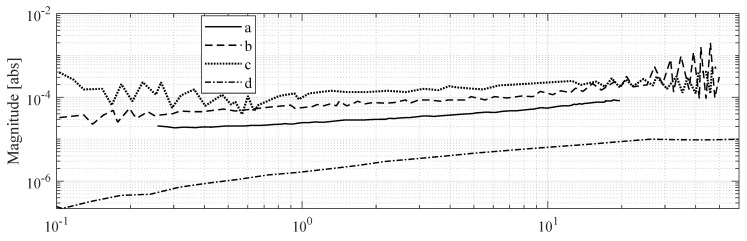
Reported frequency responses for IPMC voltage sensing according to [125] (**a**), [128] (**b**,**c**), and [133] (**d**).

IPMCs do not sustain voltage upon static deformation [106,131,132,133], although [155] may suggest otherwise. In quasi-static excitation, reports show linear behavior [97] with reading only varying from 0.125 mV to 0.135 mV between 0.5 Hz and 2 Hz at 10 mm deflection amplitude [106]. Under dynamic excitation, generated voltage increases with frequency, as was shown in random excitation experiments from quasi-static up to 20 Hz in [125], up to 40 Hz in [106], and up to 50 Hz in [128]. Figure 7 summarizes frequency response magnitudes for voltage sensing, extracted from available literature. Upon impact loading, IPMCs exhibit a damped voltage output, as the free vibrations of the sample beam decay [97]. This is well-repeatable, and has a bandwidth of hundreds of Hz.

Reported phase delays range from 90° (being essentially a velocity sensor) [102], to ∼50 [128], and to 25° [125], and decrease with increasing frequency [130,133]. Punning et al. correlated the delay with the curvature radius of the IPMC sample, i.e., the more IPMC sensor is curved, the more delayed and suppressed the signal [9,140]. Reportedly, sensitivity is higher and depends less on frequency in short and well-hydrated samples [117,125].

In experiments, IPMC voltage is amplified using voltage amplifiers (including instrumentation amplifiers) with the gain of typically below one thousand, e.g., 17.3 [124], 100 [111,125,126,127,128], 200 [154], 248 [129,129], and 270 [130]. In [106] the IPMC output voltage was measured directly with data acquisition board, without additional amplifiers. In order to use IPMC in position sensing applications, the signal conditioning should include a memory element [126], the initial position has to be known [131], and it is necessary to periodically correct for measuring error basing on external measurements [132]. Regarding the voltage offset, Park [127] argued that this offset should be corrected by subtracting the initial reading instead of using high-pass filters that distort the signal.

Noise in voltage measurements becomes dominant over signal at low bending radii due to low-voltage generation [106]. Frequency responses further show that the noise becomes stronger than the signal at above 10 Hz [125,128,133]. Several filtering techniques have been applied for noise reduction, such as bandpass filter (0.01–10 Hz) [106], high-pass filter (0.01 Hz) plus a notch filter (60 Hz) [126], and digital filters [131,132].

### 5.2. Current

**Figure 8:**
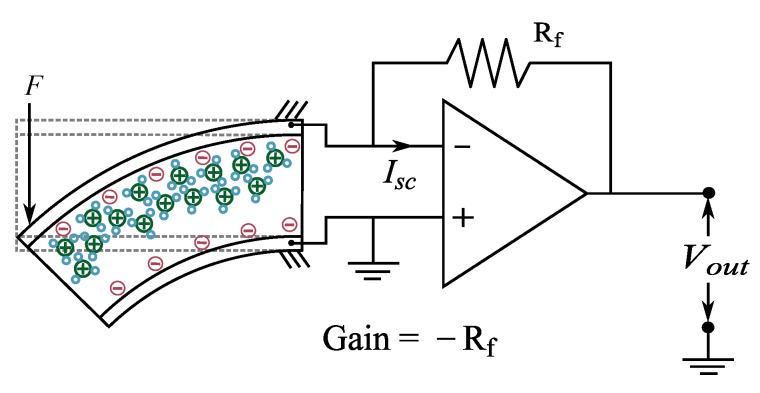
Principle implementation of IPMC current sensing.

IPMC sensing can also be realized by measuring electric current in response to bending. This case, (near-)zero voltage is maintained between the IPMC electrodes, and current that is required to maintain the zero voltage is measured [100,125]. Typically, this is implemented as an active current amplifier with virtually zero input impedance, as shown in Figure 8. Naturally, perfect short-circuit cannot be realized since wires exhibit non-zero resistance [159]. Processes behind the sensing phenomenon are the same as discussed in detail in Section 3.3.

#### 5.2.1. Reported Studies

First reports on using current measurements for sensing IPMC deformations date back to 2002, when Newbury studied the relations between the IPMC charge and tip displacement, and IPMC current and tip velocity [125]. Respective discrepancies from linear relation were attributed to the non-ideal behavior of the signal conditioning circuit [125]. In [85], Newbury and Leo built a two-port bidirectional model for both IPMC sensing and actuation, and reported that IPMC short-circuit current is linearly proportional to its velocity, with sensitivity of $10^{-4}$ As/m. Farinholt and Leo also studied the relation between IPMC current and sample tip velocity in order to validate their sensing model (see Section 3.3) [100] and reported similar correlation to the rate of displacement.

In 2006, Bonomo et al. validated their grey-box model of IPMC sensing (see Section 3.2), and reported that for optimal IPMC current sensing the IPMC hydration level should be in equilibrium with the environment, i.e., no excess water should be present in the sample [91]. Again, the model and experimental results showed that current behaves similarly to the derivative of the position, but good coherence between current and displacement measurements only occurred in a narrow range of operation frequencies [91]. In [134] authors experimentally showed that thicker polymer membranes generate higher current amplitude at frequencies below 7 Hz, and thinner membranes yield higher amplitudes at higher frequencies. Furthermore, $Li^{+}$ was also shown to be better cation choice than $H^{+}$ for IPMC sensing [134].

In 2008, Paola et al. investigated using IPMCs as vibration sensors by attaching a mass in the tip of an IPMC beam, exciting the other tip with an electromechanical shaker, and measuring the short-circuit current [21]. Results showed good agreement with model predictions, and sensitivity of 1.1 μA/g at 100 Hz. In 2013, Lei et al. used a similar design to validate their IPMC sensing model (see Section 3.3), and also reported that IPMC current shows a differentiator behavior between 10–150 Hz [107].

Hunt et al. used IPMC short-circuit current to achieve closed-loop operation of an integrated IPMC sensor-actuator design [65,82]. First a black-box [82], and later a physics-based [65] models of IPMC sensing dynamics were used to provide position feedback, and sensing dynamics was reported for three different types of IPMCs between 0.01–20 Hz [65]. Time-domain results for following step, sinusoidal, and random reference signal showed that position sensing is compromised due to integration of measurement noise [65].

Zhu et al. studied IPMC current sensing, and reported that signal amplitude depends on cation species as $Li^{+}>Na^{+}>K^{+}>H^{+}$ [118]. They observed that the magnitude of frequency response increases steadily between 0.1–10 Hz. As with [100], they observed that upon step input, IPMC current output first increases rapidly, and then decays to zero [118]. This decay was reported to vary with humidity level, and completely dry sample showed no current output at all.

MohdIsa et al. compared active sensing methods of IPMCs and reported that current sensing generally outperforms the voltage and charge sensing methods, providing higher magnitude, better coherence and less noise [133]. All sensing methods were implemented on the same material samples, and sensing dynamics of all three methods were studied in the frequency interval of 0.08–60 Hz.

#### 5.2.2. Sensing Characteristics

When step displacement is imposed at the tip of an IPMC beam, the short-circuit current response of the sample is roughly proportional to the velocity of the tip, i.e., it will first show a rapid rise, followed by rapid decay to zero, as the displacement stabilizes [85,100,118,125]. In response to step displacement, peak sensitivity of 49μAs/m has been reported (current per velocity) [100]. IPMC sensitivity was reported to be linearly proportional to sample width, inversely proportional to sample free length, and increase with the size of counter-ion species [100]. For 10 Hz excitation frequency, sensitivities of 0.5μAs/m [125], 2.4μA/mm [128], −38 dB [103], −95 dB [107], $10^{-7}$ A/mm [65], and ∼$10^{-7}$ A/mm [133] have been reported. Upon sinusoidal excitation, both linear [21] and non-linear [128] behavior of current response have been reported.

**Figure 9:**
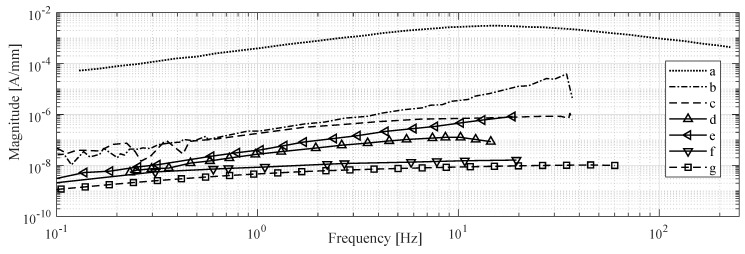
Reported frequency responses for IPMC current sensing according to [91] (**a**), [128] (**b**,**c**), [65] (**d**,**e**,**f**), and [133] (**g**).

IPMC short-circuit current has zero sensitivity to static deformations [100,133]. While it is a consensus that upon dynamic excitation the response magnitude (first) increases with frequency [65,91,103,107,118,121,128,133,160], reports disagree on the exact behavior. Figure 9 shows reported IPMC current sensing dynamics, extracted from literature. Response magnitude has been reported to steadily increase within the frequency intervals of 1–20 Hz [103], 1–150 Hz [107], and 0.1–20 Hz [65]. According to some reports, the response magnitude saturates to a constant value at approximately 10 Hz and above [65,128,133], and at 1–2 Hz and above [65,128]. It has been also reported that the response magnitude increases up to a peak frequency, and further decreases with increasing frequency, with reported peak frequencies of 20 Hz [91], 45 Hz [128], and 10 Hz [65,125].

Phase behavior shows that IPMC current response is not always proportional to the position derivative (i.e., tip velocity, requiring 90° phase), and that it does not necessarily remain constant over the investigated frequency range. For example, the phase angle has been reported to vary from 90° to −70° with center frequency (CF) of 20 Hz [91], from 45° to −150° with CF 30 Hz [107], from 80° to 20° with CF 1 Hz [65], from −165° to −280° with CF 10 Hz [125], and from 65° to 20° with CF 1 Hz [133]. In [65,103], steady decrease in phase with frequency from 90° to 20° [103] and from 120° to 40° [65] have been reported. Time delay (and thus phase) in IPMC sensing has also been reported to depend on sample geometry [100].

Measuring IPMC short-circuit current requires circuitry that maintains the virtual short-circuit condition, converts the current reading into voltage, and amplifies it. Both single-stage [100,125] and two-stage amplifier designs [65,103,107] have been exploited, and gains of $10^{5}$ V/A [100], $10^{3}$ V/A [134], $10^{4}$ V/A [128], $10^{5}$ V/A [65], and 116,000 V/A [133] have been reported.

Since IPMC current sensing exhibits low gain at low frequencies and provides no steady-state output, then using it for position sensing is complicated by weak signal at very low frequencies [134,135], and accumulation of error due to required integration action [47,65,82]. Signal-to-noise ratio also becomes poor at higher frequencies due to high amplifier gain and difficult-to-manage power network noise (50 Hz/60 Hz), e.g., above 50 Hz [128,134,135]. Bonomo et al. suggested that optimizing IPMC hydration level allows minimization of the IPMC-originated noise in measurements [91]. Despite these limitations, IPMC current sensing has been shown to produce less noisy measurement than voltage sensing [118,133].

### 5.3. Charge

**Figure 10:**
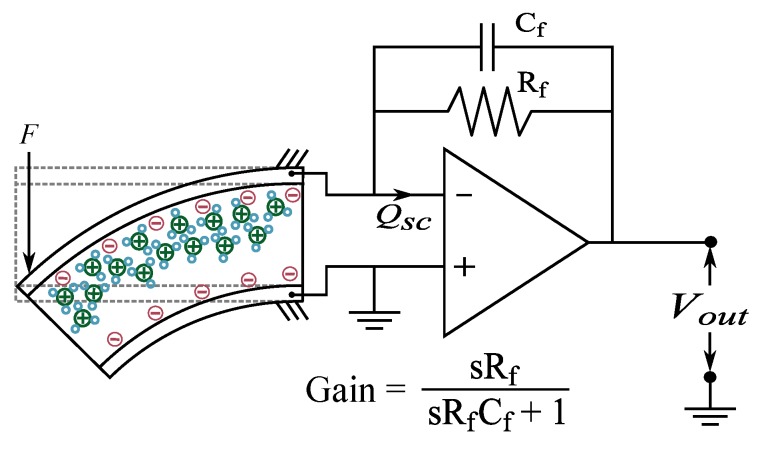
Principle implementation of IPMC charge sensing.

When IPMC is bent, ions within the polymer membrane will move and re-distribute unevenly in its thickness direction, inducing charge imbalance (and thus voltage) between the material electrodes [100,161], as discussed in Section 3.3. This charge imbalance can be measured for deformation sensing purposes using charge amplifier circuit as illustrated in Figure 10.

#### 5.3.1. Reported Studies

In 2002, Newbury characterized the dynamics of IPMC charge sensing in response to bending deformations within the frequency interval of 0.1–20 Hz [125]. It was reported that measuring charge on IPMCs exhibits better sensitivity in response to deformations than on PZT and PVDF sensors [125]. It was also lined out that the measurement circuit exhibits high-pass filter behavior and causes poor sensitivity at frequencies below 2 Hz. Similar results to [125] were reported by Newbury and Leo [89]. They characterized IPMC charge-displacement and force-voltage dynamics to validate their bidirectional grey-box model for IPMC sensing and actuation, introduced in Section 3.2.

Bennett and Leo (2004) studied charge sensing characteristics of IPMCs, and compared performance of water- and ionic liquid (EMI-Tf) solvated materials. The latter offer many application opportunities since they are electrochemically more stable and do not evaporate in air [136]. Experimental results in 1–100 Hz frequency range showed that the water-solvated IPMCs exhibit better charge-strain sensitivity, while the charge-stress sensitivities are similar. It was argued that IPMC sensing mechanism is stress-induced and not strain-induced [136]. Etebari et al. studied usability of IPMCs as shear sensors by applying harmonically oscillating shear stress to IPMC sample and measuring the generated charge [158]. IPMCs were fabricated by DAP [46], used ionic liquid for solvent, and were laminated between 2.5μm thick Mylar layers. Experiments in ∼ 45–105 Hz interval showed the peak sensitivity of 0.51 Pa/V, and a −20 dB/decade sensitivity slope, which was eliminated by correcting for the impedance mismatch between IPMC and charge amplifier. Sensitivity was shown to be influenced by temperature, while effects of vibrations and pressure were negligible [158]. Griffiths et al. studied charge-deformation characteristics of similar materials (DAP manufacturing [46], EMI-Tf ionic liquid, 25μm Kapton and 1.5μm Mylar encapsulation) with different electrode and membrane thicknesses. Investigated samples showed increasing sensitivity between DC and 20 Hz, and close-to-flat sensitivity between 20–60 Hz, while between 60–100 Hz the sensitivities showed inconsistent behavior. Electrode and membrane thicknesses were reported to highly influence the sensitivity.

Liqun et al. (2014) measured IPMC charge for force sensing purpose, and reportedly achieved 0.065 mN resolution [137]. In 2017, Gudarzi et al. studied using IPMCs for dynamic pressure sensing in a shock tube set-up [26,27]. IPMC membrane showed 1000 times higher sensitivity than the commercial piezoelectric sensor used for calibration, and it was argued to be due to larger membrane area and higher sensitivity of the IPMCs [26]. In compression and shear stress measurements, IPMC was reported to exhibit respectively 800 and 1300 times higher sensitivities than a commercial sensor [27].

In 2019 MohdIsa et al. studied IPMC active sensing methods and reported that below ∼1 Hz the charge sensing signal is weak, noisy and shows almost zero coherence to displacement [133]. Between 1–2 Hz the coherence reportedly becomes close to 1 and signal continues to improve until ∼15 Hz (amplifier cut-off frequency), after which it plateaus.

#### 5.3.2. Sensing Characteristics

Charge that is displaced by an IPMC is roughly proportional to the bending deformation [85,87,100,125] or the pressure [26] applied on the material sample. Shorter and wider IPMC samples yield higher sensitivities [125], and reported charge sensitivities to bending deformations remain in sub-μC/mm range. At 1 Hz excitations, sensitivities of 500 nC/mm [125], 300 nC/mm [89], ∼1 nC/mm [133], ∼50 nC/mm (integrating the measured current) [128], 0.622 nC/kPa (as pressure sensor) [27], and 0.2150 V/mN (force sensing) [137] have been reported. In charge sensing, IPMCs show higher sensitivity to deflections and applied forces than bending PZT and PVDF sensors, while the latter are better in voltage sensing [125].

**Figure 11:**
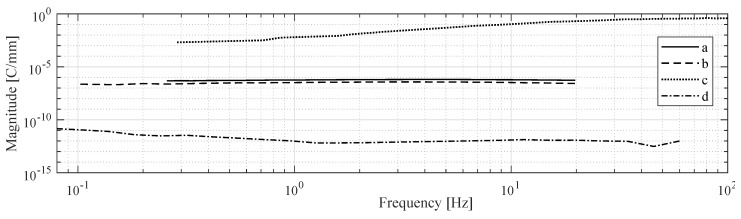
Reported frequency responses for IPMC charge sensing according to [125] (**a**), [89] (**b**), [119] (**c**), and [133] (**d**).

Figure 11 illustrates IPMC charge sensing dynamics, as extracted from the literature. It is not possible to use IPMC charge measurements for measuring static or very low-frequency deformations due to the ’memory effect’ associated with charge redistribution within the polymer membrane [89], charge leakage through IPMC internal impedance [26], and imperfections in the measurement circuitry [133]. In quasi-static operation, i.e., sub-1 Hz, IPMC sensitivity rapidly increases from zero to nearby peak sensitivity [89,119,125]. It has been argued that this is due to the filtering effect of the charge amplifier implementation, and near-flat sensitivity can be achieved when using the inverse model of the filter to compensate for its effects [125]. For frequencies above 1 Hz, good repeatability [87,89], linearity, sensitivity and reliability [26] have been reported. Between 1–10 Hz, close to constant sensitivity was achieved in [89,125], while gradual increase with frequency was observed in [119,128,136]. In [119], close-to-flat magnitude in charge-displacement sensitivity was shown in 20–100 Hz interval. According to [125], charge measurements are less noisy at high frequencies than voltage measurements. Reported phase delays of ∼0 [89,128] and ∼−170 [125] are consistent presuming reversed signal polarity, while [133] reports ∼−90 phase angle.

A principle implementation of IPMC charge sensing is shown in Figure 10, and reported studies use either single- [26,125,136] or two-stage [119,133,137] amplifiers. These circuits convert IPMC charge to voltage, and provide the required amplification. Charge-to-voltage conversion and signal amplification are achieved with the capacitor in the feedback path, and a resistor is added in parallel to the capacitor in order to prevent the amplifier from saturating due to operational amplifier’s input bias current [162]. As a result of this, the amplifier (i.e., the first stage) obtains high-pass filter effect with the cut-off frequency of $f_{c}=1/\left(2\pi R_{f}C_{f}\right)$ [89], the high frequency gain of $1/C_{f}$ [136], and significantly attenuated output at frequencies below the cut-off frequency [125,133,136]. Therefore, charge amplifier circuit design is a trade-off between gain and cut-off frequency [125]. For example, Newbury used a 460 nF capacitor and 465 kΩ resistor, resulting in 0.76 Hz cut-off frequency [125]. Charge that is produced by the IPMC can be achieved by dividing the amplifier output with the complex gain of the circuit [89,125].

## 6. Passive Sensing Methods

Passive sensing methods rely on variations in IPMC-related electrical impedance properties in response to material deformations, and require measurement circuit that also supplies power needed to detect these changes. Reported methods measure either variations in capacitances between IPMC electrodes and external metal plates [142], or variations in the impedance of IPMC electrodes [9,11,140,141]. These approaches will be addressed in the following.

### 6.1. Capacitive Sensor Element with IPMC

**Figure 12:**
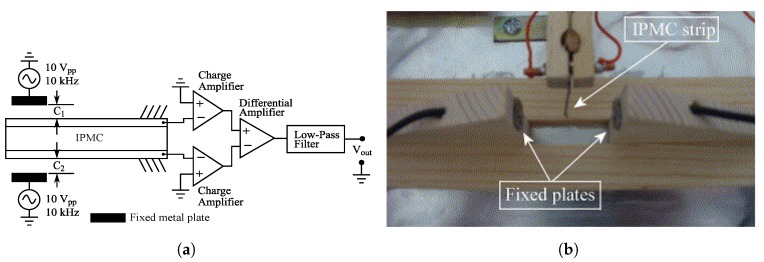
IPMC sensing method that relies on measuring capacitances between IPMC electrodes and fixed metal plates. (**a**) Schematic. (**b**) Experimental set-up (reproduced from [142], Copyright (2005), with permission from Elsevier).

In 2005 Bonomo et al. proposed a sensing method for IPMCs, where a capacitive sensor element is formed by placing an IPMC sample into an air gap between two metal plates, as shown in Figure 12 [142]. Sensing signal was created by supplying a 10 kHz sinusoidal input with 10 Vpp amplitude, and mechanical oscillations were induced by exciting the sample with a compressed air jet. IPMC tip deflection was reported to correlate with the difference between capacitances $C_{1}$ and $C_{2}$, and the circuit output showed linear relation to IPMC tip deflection, as long as the tip deflections remain much smaller than the distance between the fixed metal plates (i.e., 6 mm versus 40 mm).

### 6.2. Sensing IPMC Electrode Impedance

**Figure 13:**
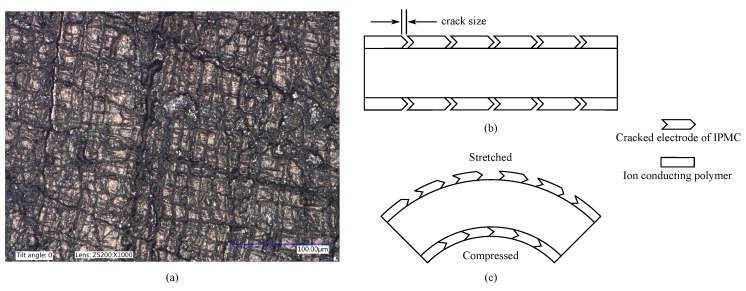
Cracked structure of IPMC metal electrodes. Bending varies the width of the cracks, i.e., they widen on the stretched side, and tighten on the compressed side, varying the resistance of the electrodes. (**a**) Microscopy image of an IPMC electrode. (**b**) Electrode structure at rest. (**c**) Electrode structure when bent.

This method is based on measuring changes in electrical impedances of IPMC electrodes that occur during bending due to stretching and compression of the thin metal electrodes [9,11,140]. This alters the size of the cracks in the electrode microstructure, as illustrated in Figure 13, further causing resistance along the electrodes to increase (stretching) or decrease (compressing) [140].

In 2007 Punning et al. reported that there exists a correlation between IPMC curvature and its surface electrode resistances, and its behavior is asymmetric, depending on whether the electrode is compressed or stretched [140]. Various constant curvatures were applied using lab glassware, and on the stretched side several times higher change in resistance were seen than on the compressed side, e.g., ∼32 Ω versus ∼4 Ω in response to 15 mm bending radius. Furthermore, Punning et al. showed that using electrode impedance variations for sensing (with 0.1 V supply voltage) produces a sensing signal that is 10–70 times stronger and less noisy than their experimental results for voltage sensing, and that static deformations can also be sensed [9]. Kruusamäe et al. (2009) studied dynamic variations in IPMC electrode resistance between 1–100 Hz, and reported noticeable variations at carrier signal frequencies of up to 20 Hz while actuating the IPMC with 0.1 Hz square wave signal of 1.5 V amplitude [141].

**Figure 14:**
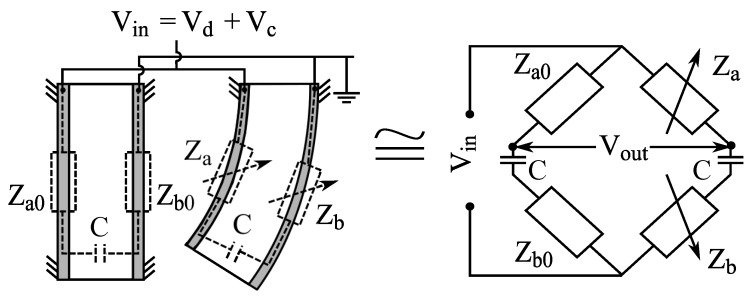
Principle of passive sensing and SSA methods that connect IPMCs into Wheatstone bridge configuration [11].

**Figure 15:**
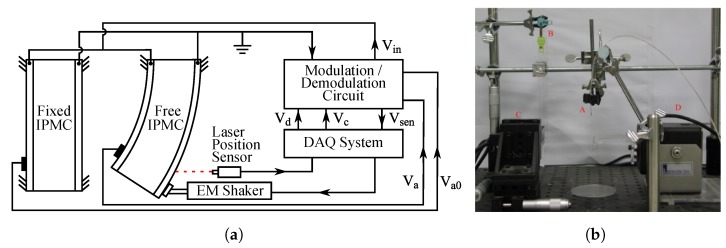
Implementation of IPMC passive sensing and SSA methods using Wheatstone bridge configuration by Fang et al. [11]. (**a**) Schematic view. (**b**) Experimental set-up, with ‘A’ deformed IPMC, ‘B’ undeformed IPMC, ‘C’ laser sensor, and ‘D’ shaker [11]. Reprinted from [11], Copyright (2010), with permission from Elsevier.

In 2010, Fang et al. used the concept from [9] to build a Wheatstone bridge from two electrically identical IPMCs, where one IPMC is bent while the other one is mechanically constrained, as shown in Figure 14. IPMC electrode resistances and through-material capacitances formed the four impedances of the bridge circuit, and variation in impedances resulted a change in the voltage reading. Proportional and polynomial models were used for position estimation. Since IPMC actuation bandwidth remains below 100 Hz, the bridge was supplied with a 1 kHz sine wave with 1 V amplitude, and sensing signal was obtained using the set-up shown in Figure 15. This solution also enabled simultaneous sensing and actuation, described later in Section 7.1. In validation experiments, the free end of the IPMC beam was excited externally, in atmospheric environment, with 0.1 Hz sinusoidal and square waves, and a sum of 0.1, 0.5 and 1 Hz sinusoidal waves, achieving position estimation error of <1 mm. Results showed that ionic liquid (BMIM-BF${}_{4}$) solvated IPMCs yield better sensitivities than water-solvated IPMCs, and authors argued that more cracked electrodes result in better sensitivity. A challenge with this method resides in spontaneous unbalancing of the bridge circuit [11].

## 7. Self-Sensing Actuation Methods

Self-sensing actuation (SSA) methods achieve sensing and actuation simultaneously within the same material sample [9,11,125,163]. Advantages of SSA methods include eliminating additional sensor weight, volume, integration and cost challenges [163,164,165,166,167]. SSA methods have been reported for a multitude of smart materials, such as piezoelectric cantilevers [168], -stacks [169] and -fibers [164], dielectric elastomers (DEA) [170], magnetorheological elastomers (MRE) [171], and carbon nanofibers (CNF) [172]. SSA methods for a wide scope of ionic electromechanically active polymers were reviewed in 2015 by Kruusamae et al. [163]. Applications of SSA include active vibration control [165,173,174,175], structural health monitoring or healing [176,177,178], system parameter identification [179], control algorithm optimization [167,179] etc. IPMCs too can be used for simultaneous sensing and actuation [9,11,12,125,144,147,163], and the challenge resides in isolating the sensing signal from the effects of actuation.

By working principal, the reported IPMC self-sensing actuation methods can be distinguished as: (1) methods that measure actuator electrode impedance (Section 7.1); (2) methods that measure impedance through IPMC actuator (Section 7.2); (3) method that measures charge on IPMC actuator (Section 7.3); and (4) methods that introduce separate actuator and sensor regions on the same IPMC sample (Section 7.4). A comparison of these methods is provided in Table 2. Simultaneous actuation and sensing has also been achieved by integrating separate IPMC actuator and sensor samples either by stacking [90,180], or by mechanically linking them together [65,82,145,181,182]. These are not considered to be SSA methods since they are not implementable on a single IPMC sample.

### 7.1. Sensing IPMC Electrode Impedance

**Figure 16:**
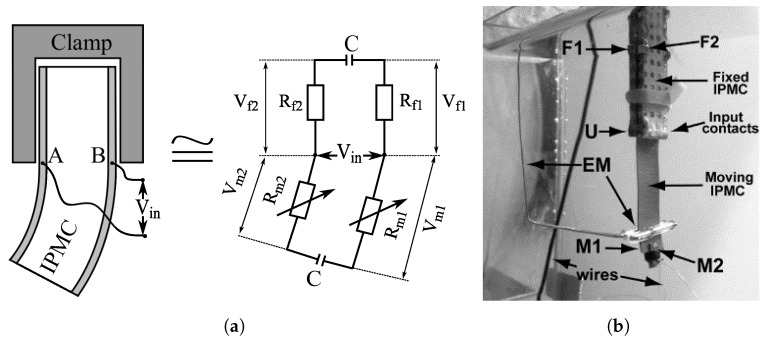
IPMC self-sensing actuation method that immobilizes half of the IPMC to provide reference impedances [9]. Surface resistances of the fixed (*R*_*f*1_ and *R*_*f*2_) and mobile half of the IPMC (*R*_*m*1_ and *R*_*m*2_) vary differently, and voltage difference between ends of the electrodes (*V*_*m*1_ − *V*_*f*1_ and *V*_*m*1_ − *V*_*f*1_) correlate with bending. (**a**) Schematic view. (**b**) Experimental set-up, reprinted from [9], Copyright (2007), with permission from Elsevier.

The working principle of these SSA methods is based on variations in IPMC electrode impedances upon deformation, as introduced in Section 6.2. IPMC actuator is connected into bridge circuit similar to Wheatstone bridge [162,183], as illustrated in Figure 16 [9] and Figure 14 [11].

Punning et al. showed that deformation-dependent surface resistance of IPMCs [140] can be exploited for sensing during actuation [9]. In their measurement configuration, one half of the IPMC actuator is fixed to provide reference impedance, and the other half is free to bend, as shown in Figure 16, resembling a Wheatstone bridge circuit of Figure 14. Actuation voltage is applied in the middle of the sample, and voltages are measured in both ends of the sample. Experiments were conducted using a 1 Hz square wave with 2.5 V amplitude for excitation. For both electrodes of the IPMC sample, voltage difference between the sample ends were demonstrated to correlate with the bending, while estimating IPMC deformation was not implemented. This method is reportedly effective for measuring both static and dynamic deformations, but the immobile half of the IPMC increases net power consumption, and the method is challenging to implement [9].

**Figure 17:**
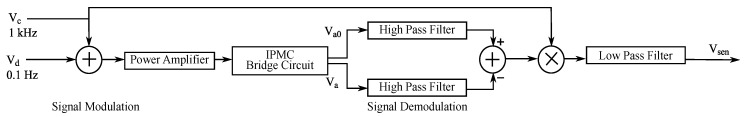
Signal processing implementation for the SSA method in [11] (see Figure 14). Low-frequency actuation voltage is modulated with high-frequency reference to produce a deformation-dependent sensing signal.

Fang et al. implemented a similar SSA method to [9] using a Wheatstone bridge type of measurement circuit [11], as previously introduced in Section 6.2, and as shown in Figure 14. In contrast to [9], a separate fixed IPMC sample was used to provide reference impedances. Sensing signal was separated from actuation voltage using a modulation-demodulation technique that was implemented as described in Figure 17. This allowed separation and measurement of the amplitude of the 1 kHz reference signal from the 0.1 Hz square wave actuation voltage with 4 V amplitude. It was reported that the average change in electrode impedance varies proportionally to the tip displacement at small deformations (at 0.33 Ω/mm in 3 mm range), and that water-solvated IPMCs (in $Na^{+}$ form) perform 15 times better than ionic liquid solvated IPMC. Limitations of this method include non-linearities from asymmetrical electrode impedance behavior (see [140]), sensitivity to dissimilarity in electrode impedances between the two IPMCs, and complexity of implementation. It was also noted that this SSA method experiences a trade-off between sensing and actuation, since larger variations in electrode impedances improve sensing, but deteriorate actuation performance [11].

### 7.2. Sensing Impedance Through IPMC

**Figure 18:**
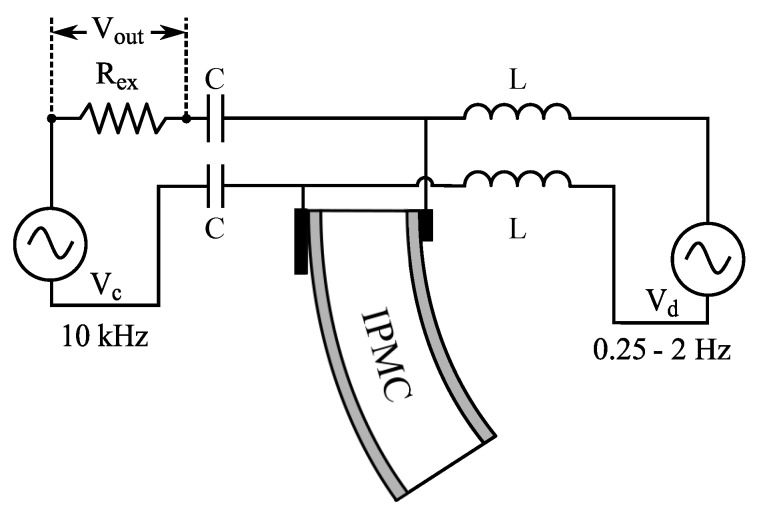
Sensing IPMC deformations during actuation by measuring the changes in through-IPMC impedance [143] and IPMC clamp contact impedance [12]. While the former method uses symmetric IPMC clamp, the latter relies on asymmetric clamping electrodes design (as illustrated in the figure) that increases impedance sensitivity to deformations. Both methods measure the impedance using a high-frequency signal that is modulated with actuation voltage.

Bakhtiarpour et al. proposed an IPMC self-sensing method that measures changes in the through-IPMC resistance, which is dominantly resistive at high frequencies [143]. Low-frequency actuation voltage (<10 Hz) was modulated with high-frequency reference signal (>1 kHz), and IPMC resistance was measured through voltage amplitude on an external reference resistor, as explained in Figure 18, as through-IPMC impedance is dominantly resistive at high frequencies [143]. Experiments at 0.5 Hz and 1 Hz actuation frequencies with up to 3.5 V amplitude demonstrated correlation between the sensing output and deformation measurements, and ability to function in the presence of external disturbances, although being less sensitive than the laser sensor. Actuation-sensing modulation was shown to affect actuation response by less than 5%.

To improve sensitivity of the SSA method above [143], Amirkhani and Bakhtiarpour proposed an asymmetrical clamp design that increases the measurable resistance variations by introducing also varying contact area (and thus resistance) between the clamp and IPMC electrode, as illustrated in Figure 18 [12]. Actuation input was modulated with a 10 kHz reference signal to measure the series resistance of the asymmetric clamping contacts and the IPMC sample (>50 Ω) through voltage on a 1Ω reference resistor. Experiments were conducted at actuation frequencies of 0.25 Hz to 2 Hz, and proportional relation between voltage and displacement was reported. Although the method is similar to [143], it is not strictly an IPMC SSA method since it relies primarily on asymmetric clamp design, which is external to the IPMC sample. Both these methods are relatively simple to realize and do not significantly affect the actuation performance.

### 7.3. Sensing Charge on IPMC Actuator

**Figure 19:**
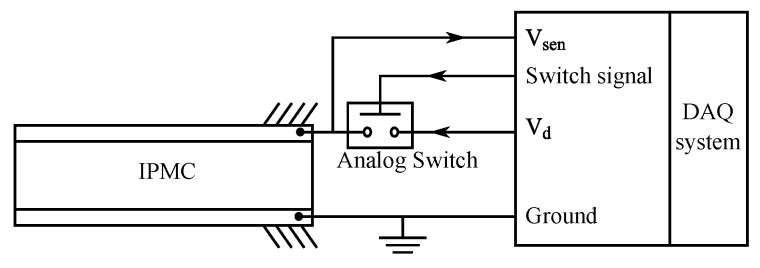
Implementation of switching between IPMC actuation and sensing for SSA [144]. Instantaneous open state is periodically introduced to the actuation voltage to measure charge stored on IPMC due to actuation voltage [144].

In 2008 Ko et al. proposed an IPMC self-sensing method that switches between actuation and sensing circuits to estimate the charge stored on the IPMC electrodes due to actuation voltage [144]. This technique is based on the assumption that IPMC charge is proportional to the deformation [100]. The method was implemented by introducing instantaneous open state to the 5 V DC actuation voltage for 20 ms in 200 ms intervals, while measuring the voltage on the IPMC electrodes, as shown in Figure 19. The voltage measurements during actuation were demonstrated to behave similarly to the position readings, while a model is required to implement position sensing. It will need to be investigated if this method is valid for position sensing also in the presence of external disturbances. In 2009, Koo et al. showed in simulations that this method could be implemented for closed-loop control of IPMC actuation [13], while experimental verification is still required.

### 7.4. Separating Actuator and Sensor Segments

These self-sensing actuation methods base on segmenting of the IPMC electrodes to separate the sample into dedicated actuation and sensing regions [10,102,125,145,146,147]. According to how the sensing signal is obtained, these reports can be further divided into active and passive types that measure IPMC-generated signal or IPMC impedance, respectively.

Active SSA methods (see Section 5) on dedicated segments of an IPMC actuator sample were first reported by Newbury in 2002 [125]. Voltage and current measurements were implemented, but proved unusable due to electrical feed-through of the actuation signal into the deformation-induced sensing signals [125]. Similar design was used in 2004 by Konyo et al., reporting that IPMC sensor voltage readings correlate with IPMC position and velocity readings [102], while actuation-sensing crosstalk was not addressed [102]. In 2007 Nakadoi et al. studied electronic interference between side-by-side sensor and actuator regions on a single IPMC sample [145], and proposed using state observers to compensate for interference. It was reported that the order of interference from actuation to sensing is approximately 1/30, while the order of ratio between sensing and actuation signals is approximately 1/1000.

**Figure 20:**
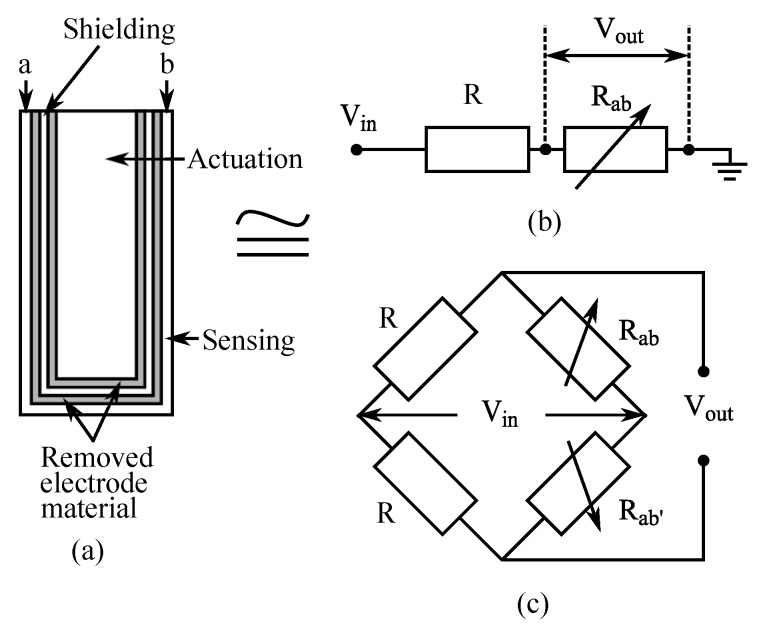
IPMC with patterned electrodes for SSA [10]. *R* represents the reference resistors, and $R_{ab}$ and $R_{ab^{\prime }}$ represent resistances from a to b on opposite faces of the IPMC. (a) Pattern implementation, where middle segment serves as the actuator, inner contour provides grounded shielding, and the outer contour serves as a resistive sensor. (b) Voltage divider measurement configuration [10,147]. (c) Wheatstone bridge measurement configuration [10,146].

**Figure 21:**
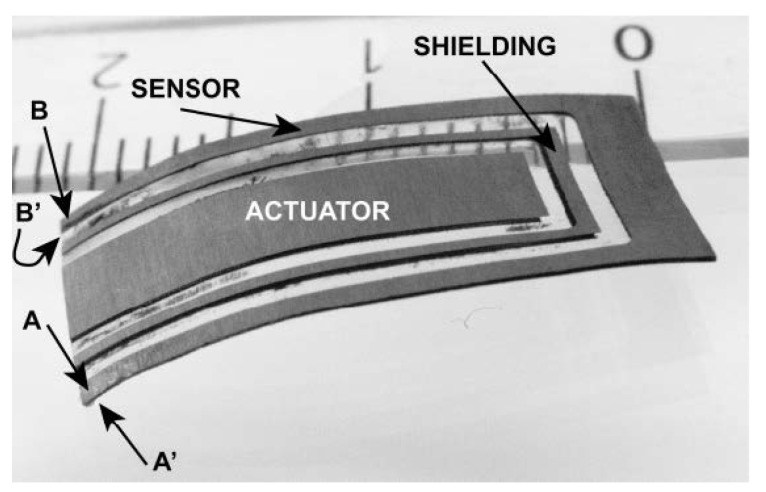
IPMC with patterned electrodes, reproduced from [10], Copyright (2010), with permission from SPIE.

Passive SSA methods on dedicated sensor segments implement methods similar to these introduced with passive sensing in Section 6. Kruusamäe et al. studied an SSA method that exploits deformation-dependency of IPMC electrode impedance by patterning separate sensor contours onto IPMC actuator sample, as shown in Figure 20 and Figure 21 [10,146,147]. Grounded shielding contour was introduced between the resistive sensor contour and the actuator area, in order to reduce disturbance from actuation to sensing signal. First, the designs were implemented using laser ablation [10] and milling techniques [10,147]. Sensor contours were connected into voltage divider configuration with 270Ω reference resistors (Figure 20b) and supplied with 1 V input, while IPMC was actuated with 1 Hz 3 Vpp input. The relatively noisy voltage divider output was reported to well agree with laser sensor reading and it was further used to identify a displacement estimation model. In [10,146] the designs were implemented using a sharp blade. Sensor contours were connected into bridge configuration with 80Ω reference resistors (Figure 20c) and supplied with 1 V input, while IPMC was actuated with 0.5 Hz 2 V amplitude input. Bridge circuit output was reported to exhibit nearly linear relation to displacement measurements. Discrepancies from linear behavior were argued to originate from asymmetric impedance change in the stretched and compressed electrodes (see [140]), and differences in sensing principles between laser and IPMC sensors [10,146,147].

## 8. Discussion

This paper reviewed the reported methods for sensing and self-sensing actuation (SSA) with IPMC transducers. It first explained the basics of IPMC materials (Section 2), next addressed modelling methods for IPMC sensing (Section 3), and then divided sensing methods into three groups in Section 4. These groups were (i) active sensing methods that make use of the electrical energy generated by the material under bending, addressed in Section 5; (ii) passive sensing methods that take advantage of changes in the IPMC electrical properties upon deformation, introduced in Section 6; and (iii) SSA methods that can be used for estimating the deformations of an actuated IPMC sample, discussed in Section 7. In the following will compare the characteristics of all these sensing and SSA methods in frequency domain, and further discuss a potential solution to overcome their performance limitations.

### 8.1. Frequency Responses of Sensing and SSA Methods

**Figure 22:**
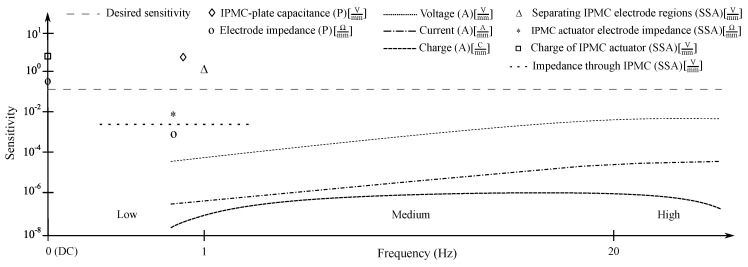
Qualitative comparison of the reported IPMC sensing and SSA methods. Shown sensitivities base on the experimental results of the respective studies. ‘A’ and ‘P’ respectively denote active and passive methods. Please note that these sensitivities are given in several different units, and therefore are not directly comparable.

IPMC sensing and SSA methods differ in sensing dynamics, and typical behavior of experimentally measured sensitivities is illustrated in Figure 22. For further discussion, the sensing dynamics will be divided into low-frequency (DC to 1 Hz), medium-frequency (1–20 Hz) and high-frequency (above 20 Hz) ranges, and addressed separately.

Under static deformations the active IPMC sensing methods do not sustain any output signal [125,133], and very high amplification is required in quasi-static measurements [65,91,125,133], due to eventual charge redistribution to its initial state [100,106,131]. This also concerns the SSA methods that separate actuator and sensor regions and rely on active sensing methods [102,125,145], which were additionally reported to suffer from crosstalk between actuation and sensing. This limitation could be alleviated by implementing a shielding segment between the actuator and sensor portions, similarly to [10,146,147]. Static and quasi-static deformations can be measured using the passive methods [11,140,142] and SSA methods that measure either the actuator electrode impedance [9,11], electrode contour impedance [10,146,147] or through-IPMC impedance [12,143]. While these methods were only demonstrated at a few discrete frequencies (see Figure 22), their usability can be assumed from the impedance-based measurement principal. The SSA method that measures charge on the IPMC actuator [144] has been validated under step excitation, but since the reported measurements durations were short (up to 4 s) then this method may be affected by the actuator’s back-relaxation effect [61,74] that did not manifest in the relatively short experiments.

In the frequency range of 1–20 Hz (see Figure 22), IPMC voltage and current sensing methods increase in sensitivity with increasing frequency [91,125,133], while charge responds with close to constant magnitude [89,125,133]. For passive sensing it has only been reported that the IPMC electrode impedances vary noticeably at frequencies up to 20 Hz (deformations not reported). In terms of SSA, the IPMC clamp contact impedance measurements have been experimentally demonstrated at up to 2 Hz [12], while other methods were not studied above 1 Hz [10,143,147].

At frequencies above 20 Hz only active sensing methods have been reported effective. Magnitude of the voltage sensing has been reported to increase with frequency until saturation [125,128,133], but also to be noisy above 10 Hz [125]. Reports disagree about the sensitivity of current sensing, describing increasing [65,103,107], saturating [65,128,133] (shown in Figure 22), and decreasing behavior after a peak frequency [65,91,125,128]. Sensitivity in charge sensing saturates [133] or decreases slightly with increasing frequency [89,125].

While characteristics of the IPMC active sensing methods agree between reports, some deviations can be observed (Section 5). These discrepancies may originate from differences in mechanical (i.e., means and nature of excitation) and electrical (i.e., signal amplification and conditioning circuits) implementations, as well as differences in environment conditions (i.e., temperature and humidity) and IPMC samples. For example, behavior of the same material type (i.e., same combination of polymer, electrode material, solvent and counter-ions) may significantly very due to manufacturing and environment conditions (see Section 3.3). IPMC self-sensing actuation methods (Section 7) and the closely related passive sensing methods (Section 6), are both much less investigated than active sensing (Section 5), but the first efforts clearly demonstrate feasibility of these concepts. Particular challenges in sensing methods vary from one method to another, but the common challenges are noisiness of the sensing signal and limited scope of experimental validation, especially in SSA. Therefore, more investigation is required to mature the IPMC sensing methods for applications.

### 8.2. Potential Solution for Improving IPMC Sensing Bandwidth

**Figure 23:**
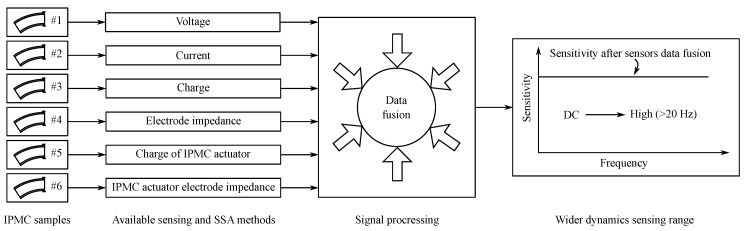
Combining sensing signals from multiple IPMC sensing methods. Individual sensing methods work reliably within their limited frequency ranges, and combining two or more methods would result in an improved deformation estimation performance.

Methods that best suit for IPMC deformation sensing depend on the frequency of interest, and there is no single method demonstrated to be effective in the entire bandwidth (see Figure 22). This would be needed, for example, in sensing IPMC actuation, where required bandwidths are between DC and 100 Hz [61,184], but high strains remain typically below 20 Hz [65,184,185]. A potential solution to overcome this limitation is combining two or more sensing methods on (one or) multiple IPMC samples, as illustrated in Figure 23. In this case the position estimation can be constructed from multiple signals of different sensing methods, such that the entire frequency range of interest will be completely covered, as illustrated by the ’desired sensitivity’ in Figure 22. Qualitative comparison of sensing methods in Figure 22 clearly suggests feasibility of this concept: SSA and passive sensing methods have been demonstrated to be useful in static and quasi-static sensing, active sensing methods perform well in dynamic sensing, and sufficient sensitivity is present in the transition frequency interval. Sensor data can be fused using known signal processing tools such as Kalman filter [186], fuzzy logic [187], neural networks [188] etc. For example, such approach has been previously reported in [189,190], etc. Castellini et al. low-pass filtered a capacitive sensor output (DC up to 500 Hz bandwidth) and high-pass filtered a PVDF sensor output (0.4−10 kHz) to tailor an almost flat force sensing response with wide bandwidth [189]. Fleming et al. combined PZT and capacitive sensor outputs using linear estimator and Kalman filter to overcome temperature-dependency in nano-scale position estimation [190].

While techniques described above address limitations of today’s IPMCs, more reliable materials are required for IPMCs’ introduction and wider use in practical sensing applications. Performance improvement is targeted in ongoing research of a multitude of research groups (Section 2), e.g., through tailoring better combinations of polymers, counter-ions and solvents, and improving repeatability of manufacturing [191,192,193,194,195]. These efforts are supported by modelling studies (Section 3), improving understanding of the relevant processes and properties. Besides IPMCs, there are several other ionic polymer transducers emerging that show similar macroscopic behavior, and may evolve faster into application-mature transducers [163,196,197,198,199,200]. Completely new transducer materials may evolve when modifying internal topology of IPMCs, and potentially combining ionic, electronic and passive transduction processes.

## 9. Conclusions

Sensing capabilities of IPMCs are expected to be useful in soft robotics, smart material mechatronics and other applications where conventional transducers are unsuitable or cannot be employed. Despite having received less attention than actuation, there have been several methods proposed to use IPMCs as deformation sensors. This paper reviewed the reports on IPMC sensing and self-sensing actuation methods and their characteristics. First, fundamentals of IPMCs were explained and modelling efforts were introduced, elucidating the sensing phenomenon. IPMC sensing methods were then divided into three categories and reviewed in terms of reported research efforts and characteristics: (i) active sensing methods, i.e., methods that measure electrical signals generated by the material; (ii) passive sensing methods i.e., methods measuring variation of impedance of circuits containing the IPMC; and (iii) self-sensing actuation methods, i.e., methods that implement sensing on the same material sample as actuation. Finally, the methods were compared in terms of their behavior in different frequency ranges, and a sensor data fusion was proposed to circumvent limitations of individual sensing methods.

It was seen that in terms of frequency responses all sensing and self-sensing method behave differently. Active sensing methods can be used for sensing in a broad range of frequencies, but are inefficient in measuring static deflections. While passive sensing methods are usable for measuring static deformations, they are noisy and have not been studied at frequencies above 1 Hz. SSA methods vary from inefficient to very promising, and more investigation is required to make conclusions over their usability. This review presented a concentrated characterization and comparison of IPMC sensing and self-sensing actuation methods. It provides a useful resource for understanding maturity of IPMC sensing methods, and selecting suitable methods for implementation.

## Figures and Tables

**Table 1:** Reported methods for sensing deformation of IPMC.

Method	Implementation	Static Sensing	Dynamic Sensing	Sensitivity (Order of Magnitude)	Remarks	
**Voltage** (Active)	Voltage and instrumentation amplifiers [111,124,125,126,127,128,129,130]	No [106,131,132,133]	Yes [106,125,128,133]	10 mV/mm [97,106,118,125,129]	Noisy at low bending radii and at high frequencies [125,128,133]. Offset has been reported in signal reading [93,106,127].	Dependent on temperature [83,84], humidity level [120,121,122,123], cation species [101,118], solvent [101,111], sample materials and geometries [24,119,138,139]
**Current** (Active)	Current amplifiers (convert current to voltage) [65,100,128,133,134]	No [100]	Yes [65,91,103,107,125,128,133]	0.01–10 μA/mm [65,125,128,133]	Outperforms voltage sensing [118,133]. Noisy at high frequencies [128,134,135]. Suffers from error accumulation [47,65,82].	
**Charge** (Active)	Charge amplifiers (convert charge to voltage) [26,119,125,136,137]	No [133]	Yes [89,119,125,128,133,136]	10–100 nC/mm [89,125,128]	Less noisy at high frequency than voltage [125]. Exhibits high-pass filter behavior [89,125].	
$Z_{\mathrm{el}}$^1^ (Passive)	Voltage divider or bridge circuit (measuring electrode resistance) [9,11,140,141]	Yes [9]	0.1 Hz [11]	10 Ω/mm [140] 0.5 Ω/mm [11]	Electrode resistances vary asymmetrically [9,140]. Feasible at carrier frequencies of up to 20 Hz [141].	
$C_{\mathrm{ex}}$^2^ (Passive)	Capacitive sensor element (requires external electrodes) [142]	0.2 Hz [142]		0.9 V/mm [142]	Sensor element consists of IPMC, airgap and external electrodes. Shown effective at IPMC deflections <4 mm [142]	

$Z_{el}$ stands for electrode’s impedance. $C_{ex}$ stands for external capacitance.

**Table 2:** Self-sensing actuation methods for IPMCs.

SSA Method	Implementation	Ref.
Actuator electrode impedance	Sensing variations in IPMC actuator electrode impedance to estimate bending (using voltage divider or bridge circuit)	[9,11]
Impedance through actuator	Modulating high-frequency signal with actuation voltage to estimate bending from impedance variations through IPMC	[12,143]
Charge on IPMC actuator	Actuation voltage is periodically disconnected to measure IPMC charge (presumed to be proportional to deformation)	[13,144]
Separate actuator and sensor segments	Patterning IPMC electrodes to create separate sensor and actuator segments on the same material sample	[102,125,145] [10,146,147]
